# Types and Patterns of Colonic Polyps Encountered at a Tertiary Care Center in a Developing Country in South Asia

**DOI:** 10.1155/2014/248142

**Published:** 2014-12-07

**Authors:** Dakshitha Praneeth Wickramasinghe, Sanjeev F. Samaranayaka, Chamila Lakmal, Sashi Mathotaarachchi, Chula Kanishka Lal, Chathuranga Keppetiyagama, Dharmabandhu Nandadeva Samarasekera

**Affiliations:** ^1^Department of Surgery, Faculty of Medicine, University of Colombo, 00800 Colombo, Sri Lanka; ^2^Professorial Surgical Unit, The National Hospital of Sri Lanka (NHSL), 00800 Colombo, Sri Lanka

## Abstract

*Purpose*. To identify the prevalence, types, and patterns of colonic polyps in a cohort of patients presenting to a tertiary care referral center in Sri Lanka. *Methods*. Endoscopy and pathology reports of a single unit from 2006 to 2013 were analyzed retrospectively. Spearman's correlation coefficient and chi-square test were used to identify correlations. *Results*. There were a total of 158 patients (M : F, 10 : 57) who had polyps encountered on colonoscopy (*n* = 1408) and flexible sigmoidoscopy (*n* = 2402) with an incidence of 4.1%. Mean age was 56.5 years (SD 16.4) and the incidence of polyps increased with age. The majority (81.6%) had one polyp. A total of 188 polyps were assessed and most were seen in the rectum (33.5%) followed by sigmoid colon (22.9%). The commonest histological type was tubulovillous adenoma (33.5%) followed by tubular adenoma (24.5%). Most polyps were benign (91.5%). There was no statistically significant correlation with age or gender with malignancy, site, or histology. *Discussion and Conclusion*. The incidence of colorectal polyps was lower than the values reported in the west. More polyps were identified in males. There was no statistically significant association between age, gender, or multiplicity and malignant change in the polyps.

## 1. Introduction

Colorectal polyps (CP) are abnormal growths of tissue projecting from the mucosa of the large bowel. They may be classified according to their morphology (sessile or pedunculated), histology (hyperplastic, adenoma, etc.), and behaviour (benign or malignant). The biggest concern is their ability to progress into carcinoma, through the adenoma-carcinoma sequence [1–3]. Accordingly, the prevalence of adenomas roughly parallels the risk of colorectal malignancy in western populations [4] where up to a quarter of asymptomatic average risk individuals will have an adenoma [5].

The data on the prevalence and distribution of polyps in Asians is limited. Data from India suggests that the prevalence of CP is about 2% [6] and they occur in relatively younger patients [7]. There is conflicting data about the commonest type [7, 8]. In Thailand, the commonest type was hyperplastic polyps [9] while it was juvenile polyps in Cameroon [10] and adenomatous polyps in Iran [11] and Saudi Arabia [12]. There is no data available on CP in Sri Lankans.

Data on the prevalence of colorectal cancer (CRC) is available in relative abundance, even in Asian countries, despite having the lowest incidence in the world [13, 14]. In Sri Lanka, the crude rate is 4 per 100,000 [13, 15] while it is approximately 2 per 100,000 in India [13, 14] and 3 per 100,000 in Thailand [13, 14].

The objective of our study was to identify the prevalence and describe the types and patterns of colorectal polyps in a cohort of patients presenting to a tertiary care referral center in Sri Lanka.

## 2. Methods

### 2.1. Population and Sample

Records of consecutive patients who underwent colonoscopy or flexible sigmoidoscopy at the Professorial Surgical Unit, Faculty of Medicine, University of Colombo, Sri Lanka, from 2006 to 2013, were analyzed. In the patients, where polypectomy or biopsy was taken, the histology reports were also analyzed. We excluded patients with polyposis syndromes.

Basic demographic data and data on the number, site, and histology were retrieved from the records.

### 2.2. Endoscopy Procedure

Colonoscopy was carried out after bowel preparation with polyethylene glycol and under pethidine and midazolam sedation. Flexible sigmoidoscopy was performed without any sedation after a sodium phosphate/sodium bisphosphate enema. Both procedures were carried out in the left lateral position.

The endoscopy was performed by a consultant surgeon or by a trainee under the direct supervision of a consultant surgeon.

### 2.3. Histological Assessment

All samples were fixed and stained with haematoxylin and eosin. They were then viewed under light microscopy by a consultant pathologist. In patients, where there was a doubt about the definitive diagnosis, the diagnosis was achieved by discussion with another consultant pathologist.

### 2.4. Statistical Analysis

Data was entered and analyzed using SPSS version 20 (IBM Corp, released in 2011, IBM SPSS Statistics for Windows, version 20.0, Armonk, NY, IBM Corp.).

All continuous data are described with mean and standard deviation (SD). Relationships between dichotomous data were analyzed using the chi-square test. Correlations between nonlinear variables were analyzed using Spearman's rank correlation coefficient.

## 3. Results

There were a total of 1408 colonoscopies and 2402 flexible sigmoidoscopies (total procedures *n* = 3810). Polyps were identified in 158 patients (M : F 101 : 57) with a prevalence of 4.1%. Mean age was 56.5 years (SD 16.4). The incidence of polyps increased with age (<20 years *n* = 4, 21–40 years *n* = 26, 41–60 years *n* = 51, and over 61 years *n* = 77). 98 polyps were diagnosed during colonoscopy (6.9% of colonoscopies) and 119 polyps were detected during flexible sigmoidoscopy (4.9% of flexible sigmoidoscopies).

The majority (*n* = 129, 81.6%) had only one polyp. Of the total 188 polyps, most were seen in the rectum (*n* = 63, 33.5%) followed by sigmoid colon (*n* = 43, 22.9) (Table 1). Of the 35 patients with polyps proximal to the splenic flexure, only 7 patients (20%) had a simultaneous polyp distal to the splenic flexure.

The commonest neoplastic histological type was tubulovillous adenoma (*n* = 63, 33.5%) followed by tubular adenoma (*n* = 46, 24.5%). The commonest nonneoplastic histology type was hyperplastic polyps (*n* = 32, 17%) followed by normal mucosa (*n* = 15, 8%) (Table 2). There was no correlation between the age and gender with site or histology.

The majority of polyps were benign (*n* = 172, 91.5%) and there was no statistically significant correlation with malignancy and age or gender (Spearman correlation).

## 4. Discussion

Our findings indicate that, in patients with CP, the commonest site was the rectum and the commonest type was tubulovillous adenoma. There was no association between malignancy and age or gender. Previous publications from India also have identified the commonest site for polyps as the rectum but the commonest histological type in their population was tubular adenoma [8]. The increasing prevalence with age, however, is well established in all parts of the world [16–19].

Autopsy studies from Europe and USA indicate that polyps are commoner in Europeans than in Asians [20] with prevalence as high as 25% or more in Europeans [17, 21] and Americans [4, 22]. Even among Asians, there is considerable variation between East Asians and South Asians, with rates closer to European/American rates being reported in East and South East Asia [23–25] though not consistent [26]. The Japanese have been identified to have rates similar to non-Latino white races [27]. However, autopsy studies have been found to have persistently higher polyp rates than endoscopy based studies [22] and the findings therefore cannot be extrapolated to endoscopic studies.

Due to the lower prevalence of polyps, the pickup rate for each endoscopic modality was also lower in our population, with much higher rates of up to 10% for flexible sigmoidoscopy and 25% for colonoscopy being reported in the USA [22]. We also failed to see a correlation between age and gender with the site and histology, though this has been reported previously in an autopsy study done in Oslo [17].

Several studies done in Europe [18], USA [4], South America [19], and Asia [8, 9] claim tubular adenoma to be the commonest form though hyperplastic polyps [10] and tubulovillous adenoma [4, 12] have been the commonest types in others. The distribution also has varied in different studies with uniform distribution throughout the colon [18], a distribution paralleling the distribution of colorectal cancer [11, 19, 28, 29], or predominance of right sided polyps [20] being reported.

Another key difference between our findings and previous authors is that we did not see a correlation between the site of polyp and age. Occurrence of proximal polyps has been found to increase with age by several authors, especially in the Scandinavian countries [9, 30] and the USA [31]. However, in India, the opposite has been reported [8].

The main limitation of our study is its retrospective nature. Another key limitation is that we have combined patients who have undergone both colonoscopy and flexible sigmoidoscopy assessments. This is because only 20% had a simultaneous polyp proximal to the splenic flexure (the usual “reach” of a flexible sigmoidoscopy). However, it is possible that our cohort of patients who underwent flexible sigmoidoscopy evaluation could have harboured more proximal polyps. Nevertheless, we believe our data would provide a reference point for assessment of Sri Lankans.

## 5. Conclusions

Our study shows that the incidence of CP increased with age. The majority had a single polyp. Most polyps were seen in the rectum followed by sigmoid colon and transverse colon. Most patients with polyps proximal to the splenic flexure will not have a simultaneous polyp distal to the splenic flexure.

The commonest histological type was tubulovillous adenoma followed by tubular adenoma and hyperplastic polyps. There was no correlation between the age and gender with site or histology. There was no statistically significant correlation with malignancy and age or gender.

## Figures and Tables

**Table 1:** Location of polyps (all polyps considered).

	Frequency	Percent
Cecum	8	4.3
Ascending colon	11	5.9
Transverse colon	26	13.8
Descending colon	23	12.2
Sigmoid colon	43	22.9
Rectum	63	33.5
Anus	14	7.4

Total	188	100.0

**Table 2:** Histology of polyps (all polyps considered).

		Cecum	Ascending colon	Transverse colon	Descending colon	Sigmoid colon	Rectum	Anal canal	Frequency	Percentage
Neoplastic	Tubulovillous adenoma	2	3	9	8	15	25	1	**63**	**33.5**
	Tubular adenoma	2	4	7	4	11	14	4	**46**	**24.5**
	Cancerized adenoma	0	0	1	1	2	3	0	**7**	**3.7**
	Villous adenoma	0	1	2	0	1	2	0	**6**	**3.2**
	Serrated adenoma	0	0	0	1	1	3	0	**5**	**2.7**
	Adenocarcinoma	0	0	0	0	0	2	0	**2**	**1.1**

Nonneoplastic	Hyperplastic polyp	2	2	6	3	9	7	3	**32**	**17.0**
	Normal mucosa	2	0	0	3	2	3	5	**15**	**8.0**
	Hamartomatous polyps	0	1	1	1	0	2	0	**5**	**2.7**
	Juvenile polyposis	0	0	1	2	1	0	0	**4**	**2.1**
	Inflammatory polyps	0	0	0	0	2	1	0	**3**	**1.6**

	Total	8	11	26	23	43	63	14	188	100
	Percentage	4.3	5.9	13.8	12.2	22.9	33.5	7.4	100	
